# Williamson on indicatives and suppositional heuristics

**DOI:** 10.1007/s11229-022-03518-z

**Published:** 2022-02-17

**Authors:** Francesco Berto

**Affiliations:** 1 Arché, University of St Andrews, St Andrews, UK; 2 Institute for Logic,Language and Computation (ILLC), University of Amsterdam, Amsterdam, The Netherlands

**Keywords:** Indicative conditionals, Acceptability of conditionals, Adams‘ Thesis, Conditional probabilities

## Abstract

Timothy Williamson has defended the claim that the semantics of the indicative ‘if’ is given by the material conditional. Putative counterexamples can be handled by better understanding the role played in our assessment of indicatives by a fallible cognitive heuristic, called the Suppositional Procedure. Williamson’s Suppositional Conjecture has it that the Suppositional Procedure is humans’ primary way of prospectively assessing conditionals. This paper raises some doubts on the Suppositional Procedure and Conjecture.

## Williamson on indicatives

In his recent book *Suppose and Tell. The Semantics and Heuristics of Conditionals* (Williamson 2020), Timothy Williamson has proposed a new defense of the view that the meaning of the indicative ‘if’ is given by the material conditional: the binary truth-functional operator with bivalent semantics, false when the antecedent is true and the consequent false, true otherwise. The view is not very popular among philosophers who work on conditionals (notable exceptions include Jackson (1987); Rieger (2013)). It has been taken as facing clear counterexamples and as unable to handle compelling data on the workings of conditionals, which are allegedly better dealt with, for instance, by probabilistic treatments (Adams 1975; Bennett 2003; Edgington 1995; Evans and Over 2004), or by modal or broadly Kratzerian ones (Stalnaker 1968; Kratzer 1986). Williamson’s intelligent, full-scale defense of a view considered dead by most researchers is likely to sparkle a fruitful debate.

According to Williamson, the putative counterexamples and data don’t actually speak against the material conditional semantics. Nor can they always be handled pragmatically, as per a traditional Gricean approach (roughly: the natural language indicatives predicted as true by the material conditional analysis, and which appear not to be, actually are true, but unassertable in the relevant contexts). The Gricean approach has been criticized by a number of authors, e.g., Edgington (1995), Bennett (2003), and Williamson (2020), 107ff, takes a number of criticisms on board.

Williamson’s strategy is innovative in claiming that the recalcitrant data have to be handled, rather than just pragmatically, epistemically, that is, as connected to a cognitive procedure—a ‘heuristic’, as he calls it—we put in place in our assessment of conditional sentences. The heuristic, like others we use, e.g., in perceptual judgments, is generally but not perfectly reliable. It delivers inconsistencies and paradoxical results when applied to cases one would not easily think about, similarly to how our heuristic for applying the truth predicate, which works fine in most everyday cases, gets us into trouble with cases like the Liar, with which philosophers obsess more than laymen (Williamson 2020[60–1]). The objections to the material conditional analysis rest on such a cognitive heuristic precisely in the cases where this is predicted to go wrong.

In this paper, I will not speak for or against the view that the propositional content of ‘if’ is adequately captured by the material conditional. I’m only interested in the Williamsonian heuristic. One reason why discussing it is important, is that it connects to influential ideas due to Ramsey, Adams, Evans and Over, and others, on how our acceptance of conditionals connects to suppositional, imaginative and hypothetical thought: a cognitive activity whose importance can hardly be overestimated. I introduce the heuristic in the following Section.

## The suppositional heuristic

The proposed heuristic is close to views endorsed by some psychologists of reasoning (Evans et al. 2003; Oaksford and Chater 2010). A main purpose of ‘if’ is to trigger, or to articulate the results of, a process of hypothetical thinking, whereby we assess the consequent on the supposition of the antecedent. What Williamson dubs the ‘Suppositional Procedure’ works thus:

First, suppose *A*. Then, on that supposition, develop its consequences by whatever appropriate means you have available: constrained imagination, background knowledge, deduction ... If the development leads to accepting *C*, conditionally on the supposition *A*, then accept the conditional ‘If *A*, *C*’ unconditionally, from outside the supposition. If instead the development leads to *rejecting* *C* conditionally on the supposition *A*, then *reject* ‘If *A*, *C*’ unconditionally, from outside the supposition. (Williamson 2020[18])

Williamson claims that the Suppositional Procedure is nothing but (what we now call) the Ramsey Test (Williamson 2020[26]). In a famous footnote, Ramsey wrote:

If two people are arguing ‘If *p* will *q*’ and are both in doubt as to *p*, they are adding *p* hypothetically to their stock of knowledge and arguing on that basis about *q*; so that in a sense ‘If *p*, *q*’ and ‘If *p*, $\lnot q$’ are contradictories. We can say that they are fixing their degrees of belief in *q* given *p*. (Ramsey 1990[155n]).

An amount of psychological as well as philosophical literature (see, respectively, Evans and Over (2004) and Bennett (2003) for classic surveys and discussions) agrees in claiming that the Ramsey Test is a high level description of at least a crucial part of what goes on in our assessment of indicatives.

Williamson takes the Suppositional Procedure as an ‘offline analogue’ of belief revision: supposing *A* is the offline counterpart of getting the information that *A*, and applying the Procedure is the offline counterpart of updating one’s beliefs in the light of the new information (Ibid). The Procedure comes with a rule which governs a variety of attitudes towards conditionals:

**Suppositional Rule.** Take an attitude unconditionally to ‘If *A*, *C*’ just in case you take it conditionally to *C* on the supposition *A*. (Williamson 2020[19])

The Procedure unpacks the cognitive process we implement to apply the Rule from right to left. Williamson’s argumentative strategy rests on a conjecture accompanying the Procedure:

**Suppositional Conjecture.** The Suppositional Procedure is humans’ primary way of prospectively assessing conditionals.^1^ (Williamson 2020[21])

The Suppositional Rule has troublesome consequences: when taken as governing attitudes that come in degrees, like intermediate credences, it leads, via plausible assumptions, to probabilistic paradoxes akin to the notorious Lewisian triviality results (Lewis 1976; Hajek 1989[etc.]). When taken as governing attitudes to logical consequences, it leads to inconsistencies. This doesn’t show that we haven’t really been following the Rule, Williamson claims. Rather, we should admit that we use inconsistent cognitive procedures to assess conditional claims (Williamson 2007[41]). This matters for the Williamsonian defense of the material conditional analysis: our cognitive practices may just be inconsistent, provided they work in the vast majority of ordinary situations; but no semantics can be inconsistent. Thus, none can take all of our judgments concerning conditionals at face value while giving the meaning of ‘if’. Next, given that we need a systematic explanation for the patterns of mistakes, ‘postulating the Suppositional Rule as a primary heuristic meets that need.’ (Williamson 2020[108])

The Suppositional Rule is about a variety of attitudes. The Suppositional Procedure, as formulated in the quotation above, concerns one fundamental attitude at issue in our assessment of conditionals: acceptance.^2^ I focus on acceptance in the next Section.

## Judgments of acceptability

What *could* count as a refutation of the Suppositional Conjecture? Insofar as it’s about a psychological heuristic, it is not refuted by examples of the conditional attitude not aligning with the attitude towards the conditional, provided such examples can be deemed infrequent, exotic, or recherché: cases of the kind philosophers trained to look for counterexamples can come up with, but which are alien to the layman’s ordinary practice. What would be needed is a widespread, systematic misalignment, possibly of a kind that can be checked experimentally. As Williamson claims: ‘the Suppositional Conjecture is a *psychological* hypothesis, which in the end must live or die by psychological evidence’ (Williamson 2020[22]).^3^

There’s reason to think that the Suppositional Rule and Procedure are violated, often enough, by the attitude of acceptance, insofar as conditional acceptance connects to judgments of probability in the natural way: we accept *C* conditionally on the supposition *A* when we assign 1, or a value that passes a threshold $\theta \in [0.5, 1)$, to *p*(*C*|*A*).

If one thinks that acceptance can come in degrees, one may also phrase the connection in terms of degrees of acceptance, with 0 marking full rejection, 1 full acceptance: one accepts *C* conditionally on supposition *A* to a degree equal to *p*(*C*|*A*). When restricted to simple indicative conditionals—conditionals with no other conditionals embedded in their antecedent or consequent—this leads straightforwardly to Adams’ Thesis (Adams 1966, 1975): the claim that the degree of acceptability of a simple indicative equals the corresponding conditional probability.^4^

We sometimes accept *C* conditionally on *A*, while *A*’s being the case doesn’t affect the acceptability of *C*, which was highly acceptable anyway. When that happens, we may not accept the corresponding conditional:

1. If Venus is bigger than Mars, then Mars is a planet.

It’s pretty common knowledge that Mars is a planet. Several people who are quite certain of that, may be uncertain of the relative size of Venus and Mars. They will accept that Mars is a planet under the supposition that Venus is bigger than Mars. Their probability for *C* conditional on *A* is or may well be 1. But they may not accept (1).

One may retort that (1) is a peculiar case, insofar as one takes its consequent as having probability 1. Conditionals with extreme antecedent or consequent probabilities are often deemed anomalous. I myself think this is wrong (Berto and Özgün 2021). Anyway, the same can happen with conditionals to whose consequents one assigns high enough but less than 1 probability:

2. If this coin is a penny, then there will be some heads in its first 50 tosses.

Say you are very confident that this coin is fair, but uncertain of what sort of coin it is. You may accept that there’ll be some heads in its first 50 tosses, conditionally on the supposition that it’s a penny: the supposition doesn’t shake your confidence that it will land heads sometimes in 50 tosses. However, you don’t assign probability 1 to this. Your probability for *C* conditionally on *A* is less than 1 and it passes your acceptance threshold.

Can one react by treating these as odd cases qua ‘missing link’ conditionals? Conditionals with no relevant connection between their antecedent and consequent, or in which the antecedent does not make a difference for the consequent, may strike us as odd (Douven 2017). One may say that in such cases we don’t even apply the Suppositional Procedure. I will discuss such a line of reply in the next Section.

Before we get there, I mention that there is *empirical*, psychological evidence that the disconnection between our accepting *C* conditionally, on the supposition *A*, and our accepting the corresponding conditional claim unconditionally, is widespread, and not a peculiarity. The experiments reported in Douven and Verbrugge (2010) focused on degrees of acceptability, understood as reasonableness to believe.^5^ Contexts (short stories) $S_i$, $1 \le i \le 30$ were given to a group of subjects, who were then asked to rate the acceptability of conditionals ‘If $A_i$, $C_i$’ in those $S_i$. The same contexts $S_i$ were given to another group of subjects, who were then asked to judge the probability of $C_i$ in $S_i$ on the supposition that $A_i$.

Now the experiments found out that people’s patterns of (degrees of) acceptance for conditionals generally don’t even *approximate* the corresponding conditional probabilities.^6^ If we agree that estimates of conditional probabilities robustly correlate to degrees of conditional acceptance, the results give some evidence that there is, often enough, little correlation between people’s acceptance of *C* conditionally on the supposition *A*, and people’s acceptance of the corresponding conditional. In particular, the acceptability ratings are in many cases significantly lower than the conditional probabilities. This ‘manifestly refute[s] Adams’ Thesis, both in its strict form ... and in its approximate form’ (Douven 2016[99]).

The conditionals that behave better with respect to the Suppositional Rule and Procedure are those in which *C* follows deductively from *A* plus background, unstated assumptions: for them, at least a high correlation was found between (degree of) acceptance and corresponding high conditional probability (Douven 2016 [p. 100]). However, the Suppositional Procedure has it that, in deriving the consequences *C* of a supposition *A*, we may use whatever appropriate means we have: we may proceed inductively, abductively, via a mixture of these, or also in imaginative ways that are not even usefully labeled as ‘inferential’—see also (Williamson 2007 [pp. 151–2]). And for the corresponding non-deductive and especially inductive conditionals, not even a high correlation was found in Douven and Verbrugge’s experiments.

Could a supporter of the material conditional analysis *cum* Williamsonian defense retort that we are dealing with conditionals that are true, but odd things to say, so that the issue is, after all, to be dealt with at the level of pragmatics? In answer to this, one should say, first, that the truth of the involved indicatives, or even just whether they can have truth values, is not what is at issue here: what is, is the workings of the Suppositional Rule, which was phrased in terms of attitudes, and of the Suppositional Procedure, which was phrased in terms of acceptance.

Second, and more importantly: the Douven and Verbrugge experiments are explicitly designed and phrased in terms of acceptability, not of assertability and its pragmatics. Acceptability as reasonableness to believe is not subject to social norms the way assertion is: one may find something very acceptable and reasonable, but inappropriate to assert in a given conversational context, e.g., because it would be off-topic, or an insensitive thing to say, or whatnot. Douven and Verbrugge anticipated possible interpretations of their results that question exactly what the subjects of their experiments thought they were being asked: ‘for instance, they might have misunderstood the questions about acceptability as asking whether it would be appropriate to contribute the given conditional to a conversation taking place in the relevant context.’ (Douven and Verbrugge 2010 [p. 310]). So they also carried out control experiments in which they (a) compared answers concerning acceptability to answers phrased directly in terms of reasonableness to believe; and (b) directly asked the participants of their main experiments questions about how they themselves had understood the notion of acceptability. Reporting on the results of such control experiments (whose detailed setup can be checked in the original paper), they concluded:

the answers do suggest that the notion of acceptability was interpreted in an epistemic sense rather than in some other sense; things that seem logical, or self-evident, or that can be taken to be true, are probably things that are reasonable to believe, though not obviously also things that it would be appropriate to contribute to a conversation. Indeed, there was no indication that any of the participants had understood ‘acceptable’ as meaning something like ‘conforming to broadly social norms governing good conversational practice.’ (Douven and Verbrugge 2010[311])

If Williamson’s Suppositional Conjecture cannot be refuted by individual recherché counterexamples because it is a ‘psychological hypothesis, which in the end must live or die by psychological evidence’; and the Douven and Verbrugge experiments, with the additional control experiment giving some evidence that the subjects of the main experiment understood well enough what they were asked, don’t count as psychological evidence against the Suppositional Conjecture; then the Williamsonian position may begin to look self-sealing: it starts to become unclear what *could* count as evidence against it.

One may come up with another line of reply, however—one which makes the issue of the distinction between acceptability and assertability moot. I come to this in the next Section.

## Missing links?

One may argue that, if the recalcitrant conditionals are mostly ‘missing link’ conditionals—conditionals with no relevant connection between antecedent and consequent—, then these pose no threat to the Suppositional Conjecture because the Suppositional Procedure just isn’t applied with them. It has been argued (Cruz et al. 2016) that the oddness of missing link conditionals is connected to the lack of a shared subject matter or discourse topic between antecedent and consequent. The mainstream way to deal with missing link conditionals is to explain their oddness pragmatically (Douven 2017). Take the pragmatically bizarre:

3. If Trump wears a wig, then there will be some heads in the first 50 tosses of this coin.

All hands may agree that this seems unassertable in most natural conversational contexts. Now, one may argue, it just doesn’t seem that we apply the Suppositional Procedure to assess (3): why would one suppose anything about Trump’s hair, develop the supposition in imagination, etc., while one wonders about the odds of this coin landing heads? We only suppose that *A*, when there is *some* connection between it and the *C* we are wondering about.

However, (2) and (3) seem to be importantly different. While it is hard (though, admittedly, not impossible) to think of a topicality connection linking antecedent and consequent in the latter, there obviously is one for the former: both are about the coin. One can wonder how the coin is like, what can happen to it. One can engage in the following procedure: supposing that this coin is a penny; assessing the likelihood that it will land heads on its first 50 tosses in the hypothetical scenario; and finding out that it’s high enough, and indeed just as high as it was for one before the suppositional exercise.

The general pattern: such cases will show up when one is unsure whether the antecedent is true; finds the consequent likely enough to be true (though one is perhaps not absolutely certain that it is); antecedent and consequent have enough overlap in topic, contextually; and it makes sense for one to engage in the suppositional exercise, to check whether the supposition of the antecedent makes a difference for the consequent. On finding out that it doesn’t, one won’t judge the conditional (very) acceptable, even if the conditional probability is high.

Take ‘Finland plays Uruguay in the next World Cup match’ and ‘Finland won’t win the World Cup’. Both are about the Finnish national team and its future doings in the World Cup. You think that, with all due sympathy, it’s quite unlikely that Finland will win the World Cup, although you are not ruling it out categorically. You are unsure who Finland is up against in the next match. You suppose it’s Uruguay, wondering whether this will make a difference for the chances of the Finnish team; develop the supposition in imagination, etc.; and conclude that Finland’s chances of winning the Cup remain just as low on this hypothesis. The probability of Finland not winning the World Cup, conditionally on playing Uruguay next, is high for you. Under the hypothesis that Finland plays Uruguay next, you accept that Finland won’t win the World Cup. You don’t come to accept the conditional:

4. If Finland plays Uruguay in the next match, then it won’t win the World Cup.

For you still deem it very unlikely that Finland will win the World Cup, and you don’t think that playing Uruguay next makes a non-negligible difference for Finland’s prospects.

Take a case in which the connection in subject matter is not engraved in the denotations of the subsentential components of the sentences, but needs a bit more context to be established: ‘My Uni runs a multi-million deficit’ and ‘I will keep my job’. It’s easy for me to provide a context: in the Spring of 2020, I was confident enough, though not completely sure, that I’d keep my academic job for a long while. (I have a permanent job and I’m doing fine; but in my country there is no academic tenure properly so called.) Then the covid-19 pandemic started, we had to shut down the campus, we began to lose money in campus revenues, etc. So I asked myself the ‘What if?’ question: I supposed my Uni would end up running a multi-million deficit, developed the supposition in imagination mobilizing my background knowledge and beliefs, etc. At the end of the suppositional exercise, I concluded that I was still just as likely to keep my job, for a series of reasons. The likelihood of me keeping my job, given the supposition, was high for me. I accepted that I’d keep my job, under the supposition of my Uni running a multi-million deficit. But the conditional

5. If my Uni runs a multi-million deficit, then I will keep my job

was not acceptable for me.^7^

Cases like (1), (2), (4), (5), have the shared feature that antecedent and consequent share topic or subject matter in natural contexts, unlike what happens with (3). And it seems to me that the corresponding suppositional processes can be, on occasion, natural enough activities to engage in: one can suppose that Venus is bigger than Mars, that this coin is a penny, that Finland will play Uruguay, that one’s Uni runs a multi-million deficit; develop the supposition in imagination, wondering about, respectively, Mars’ status as a planet, the chances of the coin landing heads, Finland’s prospects in the Cup, or one’s chances of keeping one’s job. At the end of the exercise, one can robustly accept the consequent on the supposition of the antecedent: after carrying out the imaginative exercise for a while, one stably judges the likelihood of the consequent to be quite high in the imagined scenario. One does not come to accept the conditional.

Perhaps the main point concerning cases like these, which is relevant for the Suppositional Procedure, is that one may not know or believe in advance that the likelihood of the consequent wouldn’t be affected by the envisaged truth of the antecedent; one may not have thought about the issue at all before. One carries out the suppositional exercise, and has a result: the consequent is very likely and acceptable, given the antecedent. But, one does not thereby come to accept the conditional itself. When one is unsure about an *A* with some topicality connection to a *C* one already takes as likely enough, there are lots of reasons why one may want to prospectively assess the hypothesis that the obtaining of *A* makes a difference for *C*, by making it less likely, or perhaps more likely. When, after carrying out the suppositional exercise, one concludes that *C* is just as likely conditional on *A* as it is unconditionally, *p*(*C*|*A*) is high for the subject, but the corresponding conditional can fail to be acceptable. What may be acceptable, at the end of the suppositional exercise, is some *concessive* claim, ‘Whether or not *A*, *C*’; ‘Even if *A*, still *C*’: ‘Whether or nor Finland plays Uruguay next, they won’t win the World Cup’. ‘Even if my Uni runs a multi-million deficit, I’ll still keep my job’. Concessives can take ‘even’ or ‘whether or not’ in the antecedent; ‘still’ in the consequent; and don’t generally take ‘then’ in the consequent (Douven 2016[119]), as the role of ‘then’ is precisely to rule out that *C* is the case whether or not *A* is the case (Iatridou 1993).

One helpful referee of this paper asks: what about ‘non-interference’ (as (Bennett 2003[57]) calls them) missing link conditionals, which often seem to be acceptable for one exactly when ‘the person holds *C* to be true and thinks that *A*’s being true would not interfere with that’ (Ibid.), but which don’t have an explicit ‘even’ or ‘whether or not’ in the antecedent, or a ‘still’ in the consequent? One classic example is Wayne Davis (1983)’s ‘If you open the refrigerator, it will not explode’, uttered by one who thinks that the refrigerator will not explode anyway, that is, under any circumstance. Are they just concessives without concessive-signaling words like ‘even’, ‘whether or not’, etc.? Do both concessive and Davis-conditionals differ from the other indicatives in truth conditions? Do they differ in content, in some sense of ‘content’, albeit not a truth-conditional sense? So-called *inferentialist* accounts, which take the relevance of the connection between antecedent and consequent to be part of the truth-conditional meaning of indicatives (Krzyżanowska 2015; Douven et al. 2018, 2020; Douven et al. 2021), seem to be committed to claiming that both concessives and Davis-conditionals differ in truth-conditional meaning from ordinary indicatives. This view is criticized in Lassiter (2021) and, a helpful referee of this paper suggests, the results in Cruz et al. (2016) may also be taken as speaking against it.

I can be neutral on this, however, for the purposes of discussing the Suppositional Conjecture and Procedure. For whatever the answers to these tangled questions, non-concessive, non-missing-link conditional sentences generally differ in acceptability from both concessives and Davis-conditionals, whatever the truth-conditional meaning of these. And acceptability, not truth-conditional propositional content, is what is at issue with the Suppositional Conjecture. Williamson claims that the Suppositional Procedure operates on sentences, rather than on the propositional contents they express (Williamson 2020[17-18]). The heuristic is at work at the level of verbal reasoning, and verbal reasoning operates for him on things that are more fine-grained than truth-conditional contents taken, e.g., as sets of possible worlds. As he says:

The heuristic fits in wherever verbal reasoning fits in. [...] For these purposes, a psychologically realistic theory of verbal reasoning will have to treat it as operating over structural mental representations such as interpreted sentences, not over bare coarse-grained propositions. [...] [T]he fine-grained dynamics can only be properly understood at a level where sentential structure has not been left behind. (Williamson 2020[24]).

Now ‘If *A*, then *C*’ is normally acceptable precisely when ‘Even if *A*, still *C*’, or ‘Whether or not *A*, *C*’ isn’t. And Davis-conditionals seem to have acceptability conditions that match those of concessives, whether they differ in meaning or not. One who accepts ‘If you open the refrigerator, it will not explode’ in a situation in which one thinks it will not explode under any circumstance, will accept precisely the concessives ‘Whether or not you open the refrigerator, it will not explode’, or ‘Even if you open the refrigerator, it will not explode’. And one will reject the addition of a ‘then’ before the consequent: ‘If you open the refrigerator, then it will not explode’. For such an addition would convey the idea that ‘If you don’t open the refrigerator, it will not explode’ is *not* acceptable for one; whereas, in the context, it clearly is.

Finally, only some conditionals uttered in real life have the acceptability conditions of concessives or Davis-conditionals, and possibly only a minority of them do: even lacking statistics, people with very different views on missing link conditionals and concessives agree that conditionals whose antecedent *does* make a difference for the consequent are the default in English: see e.g. Skovgaard-Olsen et al. (2016); Douven (2017); Lassiter (2021). Insofar as the Suppositional Conjecture is advanced as one that applies to indicative conditional sentences in general, it’s not enough for it to work fine with concessives or Davis-conditionals only.

## Conclusion

It may be, then, that our ‘primary way of prospectively assessing conditionals’ is not *quite* the Suppositional Procedure but rather some fixing of the Procedure, which adds to it some constraint linking antecedent and consequent: in order for us to accept ‘If *A*, then *C*’, it is necessary but not sufficient that we accept *C* on the supposition *A*; in addition, the supposition must, in some sense, make a relevant difference for the truth or likelihood of *C*. One would need to make precise the notion of a conditional antecedent ‘making a relevant difference’ for its consequent. Relevance for conditionals, however, is one of the most elusive concepts, and very different treatments of it have been proposed: (non-classical) logical, as in relevant logics (Dunn and Restall 2002); probabilistic-evidential (Douven 2016); based on default-and-penalty hypotheses (Skovgaard-Olsen et al. , 2016); on causal connectedness (Schulz and van Rooij 2019); on evidential support (Crupi and Iacona 2020); on coherence relations between discourse clauses (Lassiter 2021); or on yet other notions. How to fix the Suppositional Procedure to take relevance into account seems to be an open issue.

## Footnotes

1 We assess prospectively when we don’t know that the antecedent is true (Williamson 2020[17]). (When we do, we’d better test the consequent directly!) We can assess prospectively also without applying the Procedure, typically because we rely on someone’s testimony, but that’s a secondary or derivative, if widespread, way (Williamson 2020[20-1]).

2 And its opposite: rejection. Some philosophers, e.g., Priest (2006), dispute the view that rejection is reducible to the acceptance of negation. This is immaterial to our discussion.

3 Williamson admits that his book ‘does not contain much discussion of experimental data’ (Williamson 2020[22]): it presents a theoretical model of suppositional thinking, and of how it connects to our assessment of conditionals. Williamson claims that we should understand the model before testing it (Ibid.).

4 Adams’ Thesis is sometimes, but should not be, confused with Stalnaker’s Hypothesis (Stalnaker 1975), also called ‘the Equation’ by authors like Edgington and Bennett, and which has it that the probability of an indicative equals the corresponding conditional probability. Lewis’ and others’ aforementioned triviality results (Lewis 1976; Hajek 1989) are often taken as showing that the Equation can’t be quite right. However, it enjoys considerable empirical support in the literature. We’ll soon see how things go with Adams’ Thesis.

5 Or, as reasonableness to have an attitude functionally close enough to belief, if, as non-propositionalists like Edgington (1995), Adams (1998), Bennett (2003), etc. think, indicatives don’t express propositions and cannot generally have truth values. Then they may not be believable, strictly speaking, insofar as to believe something is to believe it to be true. But they can still be acceptable, and acceptability works in a way close enough to believability even if it needs to be disentangled from the latter, to some extent, for non-propositional indicatives. One may say that if an indicative doesn’t express a proposition, then accepting it is, or at least requires, being disposed to do certain things with it, which align to what one is disposed to do when one believes something that does have a truth value. E.g., if one accepts ‘If *p*, then *q*’ and one believes that *p*, one is prone to believe *q* as well. This function of acceptable conditionals as inference tickets is stressed by non-propositionalists: see e.g. Bennett (2003), Ch. 8.

6 As a helpful referee of this paper mentioned to me, results analogous to the Douven-Verbrugge experiments are to be found also in the influential Skovgaard-Olsen et al. (2016).

7 In case you are curious: my Uni did run a multi-million deficit in 2020. And I still have my job.
